# The Horizontal Root Fractures. Diagnosis, Clinical Management and Three-Year Follow-Up

**DOI:** 10.2174/1745017901814010687

**Published:** 2018-09-28

**Authors:** Roberto Lo Giudice, Angelo Lizio, Gabriele Cervino, Nicita Fabiana, Puleio Francesco, Pietro Ausiello, Marco Cicciù

**Affiliations:** 1 Department of Clinical and Experimental Medicine, Messina University, Messina, Italy; 2 Department of Biomedical and Dental Sciences and Morphofunctional Imaging, Messina University, AOU Policlinico “G. Martino” *Via* C. Valeria 98100, Messina, Italy; 3 Department of Neurosciences, Reproductive and Odontostomatological Sciences, Naples Federico II University, Naples, Italy

**Keywords:** Dental trauma, Root fracture, Splints, Periodontal inflammation, Pulpal pathology, Alveolar bone

## Abstract

**Objective:**

The aim of this retrospective analysis is to describe and to evaluate the middle third horizontal root fractures, long term clinical management results and to estimate the effect of treatments factors upon healing and survival rate.

**Methods:**

Our clinical study included 42 patients presenting a middle third horizontal root fracture in permanent dentition. For each patient at t_0_ the parameters recorded were: diastasis, mobility, sensibility, periodontal inflammation, pulpal pathology, associated fracture and dislocation of the coronal fragment. The follow-up was performed after 6 (t1), 12 (t2) and 36 (t3) months after the trauma, both clinically and radiologically. Clinical examination, vitality tests and a radiological evaluation (periapical x-ray) were performed.

**Results:**

At t_0 _ it was observed: diastasis (14, 3%), mobility (28, 6%), thermal sensibility (61, 9%), periodontal inflammation (4, 8%), pulpal pathology (38, 1%) and dislocation of the coronal fragment (47, 6%) of the patients. The treatment plan started with the coronal fragment repositioning and the blockage (splint) with the adjacent teeth in 47, 6% of cases.

A root canal treatment was performed at t_0_ in 52,4% of the fractured teeth. Statistical analysis showed the highest level of significance between pulpal lesions (t_0_) and associated fractures. The mobility, sensibility and pulpar lesions parameters, showed a reduction in relation to the follow up timing, with a main variation remarkably evident between t_0_ and t1. The ratio between observation time and the presence of diastasis was statistically significant.

**Conclusion:**

The analysis of the clinical results exhibits the high success rate of a conservative approach in the treatment of teeth fractured in the middle third of the root.

## 1 INTRODUCTION

A multidisciplinary approach is clinically considered necessary to manage dental trauma [1]. Clinical investigations in children and adolescents indicate that a rate of 16% -30% of these individuals has suffered of dental trauma at least once [2]. Root fracture is a very important complicating factor associated with [3]. Root fractures are lesions involving dentine, cementum and pulp, associated with injuries of the periodontal ligament and the alveolar bone; so the reparation of these damages necessitates a multi-tissue regeneration [4]. Among all dental injuries, they are uncommon, comprising nearly 0.5-7% of traumas that occur in permanent dentition [5-7]. The highest incidence range of roots fractures is recorded in male patients during the second decade of life [5, 6]. Horizontal root fractures mainly affect the maxillary anterior region. Childhood obesity, horizontal overlap, upper central incisor protrusion and insufficient lip closing are considered as individual predisposing factors [8]. The location of the fracture line and the degree of dislocation of the coronal segment are the main parameters of the horizontal root fractures classification [9].

The middle third and the apical third of the root of maxillary incisors are predominantly affected (respectively 57% and 34%, usually observed in fully erupted permanent upper incisors with closed apices and a completely formed root [10]. Root fractures are characterized by a separation of an apical segment, not usually displaced, and a coronal segment, which is often variously displaced. After the impact, pulp and the other damaged tissues start healing process. It depends on patients age, tooth mobility, degree of root formation, location of root fracture, diastasis of fragments and time between trauma and treatment [7, 11, 12]. Andreasen *et al.* [8], described 4 categories of root fractures healing:

- Healing by hard tissue union
- Healing by interposition of connective tissue
- Healing by interposition of bone and connective tissue
- Non-healing (interposition of granulation tissue)

Horizontal root fractures in the apical and middle third are not always symptomatic, and can be incidentally identified during routine examinations. In these cases, treatment is often non required and spontaneous healing is frequently reported [13-15]. In the other cases, the patient can report pain during chewing. The tooth can present increased mobility and bleeding from the periodontal ligament [8, 16]. Pulp necrosis of the displaced coronal segment occurs in about 25% of cases. Pulp necrosis of apical segment is rare [17-19] . If mobility and displacement of coronal segment are absent, Görduysus *et al*., suggest that patient may not require dental treatment [17]. The diagnosis is based on radiography and observation under magnification. In RX intraoral digital exam, the fracture line is revealed by a radiolucent line, separating the displaced coronal fragment from the apical one [20].

Sometimes the radiographic exams are limited by the position of the fracture fragments that is not properly detectable by a 2D imaging technique [14]. The American Academy of Pediatric Dentistry suggests to perform radiographic examination using different projections and angulations (periapical with 90° horizontal angle, occlusal view and periapical with distal or mesial angulation) [21]. CBCT could allow a 3D-examination of root fracture, providing additional information for assessing the prognosis, but its use must not be considered routine [22]. The literature underlines that the main treatment choice is directed to reposition and stabilize the coronal fragment using a semi-rigid or rigid splint to the adjacent sound teeth, possibly under magnification [7, 23-25]. The tooth must be checked for the following months to evaluate the pulp vitality. In case of pulpal necrosis an endodontic treatment should be carried out [26]. The pulp necrosis can involve the coronal fragment or both the coronal and the apical fragment.

In relation with the lesion topography, the endodontic treatment can involve both fragments or only the coronal fragment followed by the surgical removal of the apical fragment [27, 28]. Furthermore, Celikten *et al*., suggest as possible treatment option the extraction of the coronal fragment, endodontic treatment and orthodontic extrusion of the apical fragment [23]. The aim of the retrospective analysis is to evaluate the effect of treatments upon healing and survival rate of teeth affected by horizontal root fracture.

## 2 METHODS

**Fig. (1):**
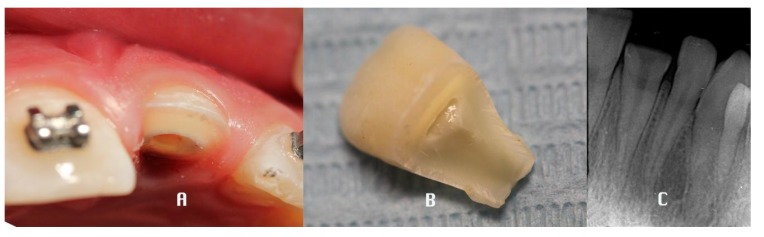
Baseline: Coronal and root middle third fractures of 2.1 **A**) Oral view **B**) Coronal fragment **C**) Intraoral Rx.

**Fig. (2):**
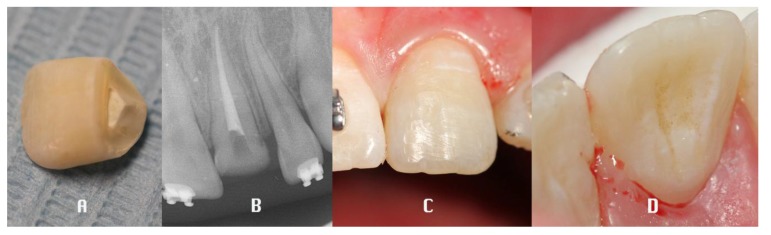
Treatment plan: **A**) Coronoplasty of the coronal fragment **B**) Endodontic treatment **C**) buccal view of the repositioned coronal fragment **D**) palatal view of the repositioned coronal fragment.

**Fig. (3):**
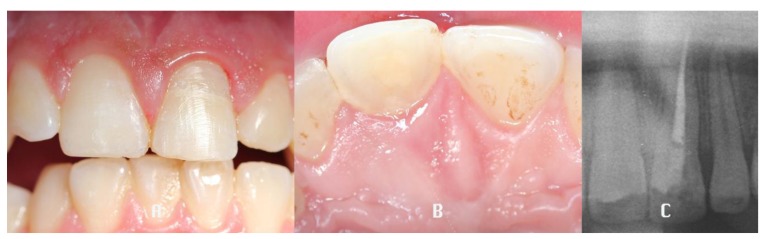
Six months follow-up **A**) Buccal view **B**) Palatal view **C**) Intraoral Rx.

**Fig. (4):**
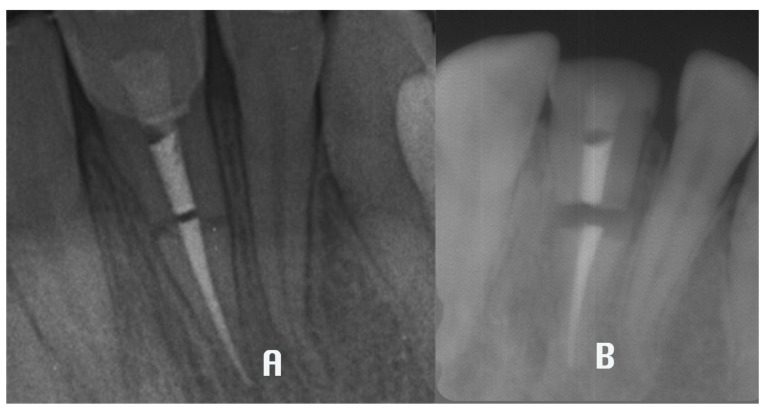
Rx follow-up: Root fracture line widening **A**) 12 months follow-up **B**) 36 months follow-up.

A retrospective clinical study was performed between 2009 and 2016 in the Department of Dentistry at the University of Messina. The analysis included 42 patients (27 males, 15 females), with a mean age of 28.24 years, presenting a middle third horizontal root fracture in permanent dentition due to traffic accident, classified by WHO as A (Injuries to the hard dental tissues and the pulp) #.7 (Root Fracture - N 502.53 - fracture involving dentin, cementum, and the pulp.) [29]. For each patient a medical history was collected and a clinical examination through inspection, palpation and percussion, vitality tests and a radiological evaluation (periapical x-ray), was performed (t_0_). The parameters recorded were: diastasis, mobility, sensibility, periodontal inflammation, pulpal pathology and the presence of associated fracture and dislocation of the coronal fragment. (Fig. **1**) The treatment plan was decided depending on the single clinical situation, performing the repositioning of the coronal fragment, with a semi-rigid splint of composite resin to the adjacent teeth and the root canal treatment in case of pulpal involvement. (Fig. **2**) The follow-up was performed at 6 (t_1_), 12 (t_2_) and 36 (t_3_) months after the trauma, both clinically and radiologically. (V. Figs. **3**, **4**) Statistical analysis was performed as follows.

The age was the only numerical variable and it was expressed as mean, standard deviation, median, minimum and maximum; the categorical variables were expressed as numbers and percentages. The non-parametric approach was used because of the low sample size. The Cochran test was applied in order to evaluate the existence of significant differences for the presence of diastasis, inflammation, mobility, sensitivity and pulpal lesions in four different times (basal, 6, 12 and 36 months). The Mc Nemar test was estimated to perform statistical comparison between two consecutive times for each parameter. In order to evaluate the association between sex, fracture, dislocation, repositioning and splint with presence/absence of diastasis, inflammation, mobility, sensitivity and pulpal lesions (for each time), the Pearson’s P square was applied; alternatively, the exact Fisher test was estimated**,** when a frequency in the contingency table was less than 5. *P*<0,050 two sided was considered to be statistically significant. Statistical analyses were performed using *SPSS* 17.0 for Window package.

## 3 RESULTS

The root fractures mainly involved: 1.1 (52,38%); 2.1(38.09%); 4.1 (4,76%), 1.4 (4,76%) (Table **1**). In 38,1% of cases there were coronal fractures associated.

At the first clinical evaluation (t_0_) the diastasis was observed in 14,3%, mobility in 28,6%, thermal sensibility in 61,9%, periodontal inflammation in 4,8%, pulpal pathology in 38,1% and dislocation of the coronal fragment in 38,1% of patients. Only in two cases (4,8%) a sign of periapical phlogosis was recorded (Table **2**).

The first step of the treatment plan was the repositioning of the the fractured coronal fragment and the splint with the adjacent teeth in 47,6% of cases. In 52.4% of cases any splint was necessary due to the absence of fragment mobility. A root canal treatment was performed in the first phase in 52,4% of the fractured teeth.

Furthermore, due to pulpal pathology, endodontic treatment was performed in two cases at the 6 months follow up and in other four cases at the 12 months observation. At the 36 months follow up, 28,6% of the teeth wasn’t endodontically treated. In one case the treatment plan consisted of extraction of the tooth because of diastasis and mobility (Table **3**).

The statistical analysis of each clinical parameter evolution is shown in Table **4**.

Between t_0_ and t_1_ the frequency of diastasis decreased from 14,3% to 0%, it remained stationary between t_1_ and t_2_ and it increased at t_3_ (9,5%).

The Cochran test (*P* < 0,050) shows the significativity (*P* = 0.013) of the ratio between observation time and the presence of diastasis. Using the Mc Nemar test in order to value the pair control (used each time where the variable is binary) only the first comparison (T_0_-T_1_) is significative (*P*= 0.031).

The mobility, sensibility and pulpal lesions parameters, show a statistically significative reduction in relation to the follow up timing, with a remarkably evident main variation between t_0_ and t_1_, confirmed by the Cochran and McNemar tests.

The pulpal pathology does not show any statistically significant differences related to the observation time.

The frequency of mobility is reduced significantly from 28,6% (t_0_) to 0% (t_1-2_) (*P*<0.001). At 36 months (t_3_) is shown the presence of 1 case of mobility (2,4%), but compared with McNemar test is not significant.

The sensibility parameter shows a significant decrease in relation to observation time, as it is evident in Cochran test (*P* < 0.001). The percentage at the first observation is 61,9%, at 6 months and at 36 months is 0%. At 12 months sensibility has been recorded in the 9,5% cases.

Mc Nemar test for the pair comparison shows how only the first correlation (0-6 months) is statistically significant (*P* < 0.001), while the others (6-12 months and 12-36 months) are not.

The gingival inflammation is present in 4,8% of traumatized teeth at t_0_ and t_1_, in 14,3% at t_2_ and in 9,5% at t_3_. Cochran test does not reveal the existence of statistical significance in the relationship between this parameter and the observation time.

The presence of pulp injury was diagnosed in 38.1% of patients at t_0_, in follow-up after 6 and 12 months it was found in an additional 4.8% of cases, while at 36 months it was not encountered in any ulterior cases. The Cochran test does detect the existence of a high significance (*P* < 0.001) including 4 observation times. The Mc Nemar test allows us to identify that the first in pairs comparison (t_0_–t_1_) is the only significant one (*P*=0.001).

The association data between each specific parameter described and other variables (sex, associated fracture, fragment repositioning, splinting procedure, dislocation of the coronal fragment) assessed with Pearson’s test and with exact the Fisher test showed the highest levels of significance between the parameter pulpal lesions at t_0_ and the presence of associated fractures (*P* < 0.001).

The 12-month periodontal inflammation parameter has significant association with associated fracture (*P*=0.038), the repositioning (*P* = 0.010) and splinting procedures (*P* = 0.010).

## 4 DISCUSSION

In the present retrospective analysis, middle third horizontal root fractures were observed in 95,23% of cases in maxillary and mandibular incisors. The most relevant factors in conditioning the therapeutic success are the reposition of the fragment displaced, the immobilization of the parts and the presence of pulpal pathology. In our study in 50% of cases the treatment plan consisted in a fragment repositioning followed by a flexible splint, no treatment was performed in the other 50% of cases The fracture diagnosis doesn’t always determine the necessity of performing a root canal treatment [13, 14]. According to IADT guidelines, our findings suggest that, if the pulp necrosis does not occur, the root canal treatment is not necessary [30]. Only in one of the 42 analyzed cases, after 36 months from the therapy, tooth extraction was required because of diastasis, mobility and phlogosis. The healing of the horizontal root fractures without treatment is presented in many reports with a rate superior to 80% [7, 15]. Makowiecki *et al*., reported a case, found incidentally on a routine radiographic examination, maintaining the pulpal health up to 17 years after root fracture [15]. In studies with large series of intra-alveolar root fractures Andreasen *et al*., and Cvek *et al.,* reported a healing rate by hard tissue fusion of the fragments respectively of 30% and 33% [11, 31]. The pulpal healing, in case of laceration, is determined by a revascularization process and could result in coronal pulp space calcification [31]. In case of absence of pulpal damage, the healing process can result in a hard tissue barrier between the fragments [31]. However, depending on the lesion characteristics, other tissues than bone, as fibrotic or granulation one can grow in the interface between fracture fragments [4, 6]. The biological process of pulp vitality maintenance involves odontoblastic and cementum cells activity. In the absence of bacterial leakage and infection, the pulp response depends on the integrity of pulpal tissue [7, 32]. Intraoral x-ray and pulpal vitality tests are necessary to detect the fracture and evaluate the pulpal status. However, many AA underlined poor accuracy of the intraoral X-ray. In fact, the presence of neighboring structures and little or no separation of fragments, could affect the correct diagnosis. Moreover, the X-ray beam and the fracture line aren’t always on the same plane [15, 33-35].

According to IADT’s Guidelines, the pulpal response should be evaluated after the trauma and up to three months later. Due to the non-reliable response to the vitality tests, endodontic treatment should be planned if after 3 months the electrometric or thermal pulp testing is negative and/or if a radiolucency is detected [30]. Our data show how in T_0_ in one case (39.09%), an endodontic treatment was performed due to the evidence of pulpal pathology. The statistical analysis underline how a high level of significance is present when analyzing the relationship between pulpal lesion and presence of associated fractures. The evidence of pulpal pathology required an endodontic treatment even 6 and 12 months after the trauma. Andreasen *et al.*, [11], found that the mobility and dislocation of the coronal fragment and fragment diastasis are two of the most important parameters in healing of the horizontal fracture and in the occurrence of pulpal necrosis. When evaluating the modification of the clinical parameters examined, the statistical analysis shows how the diastasis is significantly reduced at T1, but even if it is increased after 36 months this is not statistically significative. According to Soares Andrade *et al*., in the presence of diastasis, the repositioning of the fragments increases the frequency of healing, particularly in mature teeth [36] Andreasen *et al*., stated that splinting procedure influence pulpal and hard tissues healing between fracture fragments [19]. In several studies it is reported that a slightly flexible fixation is the best treatment to obtain a natural healing and to preserve pulpal vitality [10, 35]. In middle root fracture the ideal splinting period to achieve a good healing and to gain a long term stability of the fragment is 3-4 weeks [21]. Overcoming this timing would not result in any prognosis improvement [21, 37, 38]. From the analysis of our cases it is evident how the 2.38% presence of mobility in the samples at t_3,_ is compatible with the preservation of the tooth. We observed that the marginal periodontal inflammation will last through time and it is connected to the presence of associated fractures and to the repositioning and splinting procedures, that may produce root resorption [39]. Moreover, the thermal sensibility of the non-endodontically treated teeth will significantly reduce over the time [40].

## CONLCUSION

The analysis of the clinical results exhibits the high success rate of a conservative approach in the treatment of teeth fractured in the middle third of the root. Just in one case extraction of the involved teeth was necessary after 36 months. In 50% of cases endodontic treatment was immediately performed due to a pulp exposure caused by the fracture itself. In a low percentage of cases a late root canal treatment was performed because of pulpal and periapical complications.

A careful monitoring of all the clinical parameters involved especially mobility, diastasis and pulp status is confirmed to be crucial for a correct and conservative approach. The immediate treatment of the intra alveolar root fracture with the severely displaced coronal fragment was important for the good prognosis, aesthetical results, and long term success.

## Figures and Tables

**Table 1:** Incidence of teeth involved in root fractures.

**Tooth Involved in Fractures**	**%**
**1.1**	52,38
**2.1**	38,09
**4.1**	4,76
**1.4**	4,76

**Table 2:** Evolution of parameters at T^0^-T^1^-T^2^-T^3^.

**Parameters**	**T^0^**	**T^1^ (6 Months)**	**T^2^ (12 Months)**	**T^3^ (36 Months)**
**Diastasis**	14,3%	0%	0%	9,5%
**Mobility**	28,6%	0%	0%	2,4%
**Sensibility**	61,9%	0%	9,5%	0%
**Periodontal Inflammation**	4,8%	4,8%	14,3%	9,5%
**Pulpal Pathology**	38,1%	4,8%	4,8%	0%
**Dislocation of the Coronal Fragment**	47,6%	0%	0%	0%

**Table 3:** Treatments performed at T^0^-T^1^-T^2^-T^3^.

**Therapy**	**T^0^**	**T^1^ (6 Months)**	**T^2^ (12 Months)**	**T^3^ (36 Months)**
**Fragment Repositioning And Splint**	47,6%	0	0	0
**Root Canal Treatment**	52,4%	4,8%	0	2,4%
**Extraction**	0	0	0	4,8%

**Table 4:** Non parametric statistical analysis of the parameters.

**Parameters**	**Cochran Test**	**Mcnemar Test**	**Pearson Test** **(frequency > 5)**	**Fisher Test** **(frequency < 5)**
**Diastasis**	0.013	T^0^-T^1^ = 0.031 T^2^-T^3^ no significative		Diastasis & Repositioning (T^0^) = 0.007 Diastasis & Splint (T^0^) = 0.007
**Mobility**	0.000	T^0^-T^1^ = 0.000 T^2^-T^3^ no significative		Mobility & Dislocation (T^0^) = 0.000 Mobility & Repositioning (T^0^) = 0.000 Mobility & Splint (T^0^) = 0.006
**Sensibility**	0.000	T^0^-T^1^ = 0.000 T^1^-T^2^ no significative T^2^-T^3^ no significative		
**Inflammation And Periodontal Pathology**	No significative	No significative	Inflammation & fracture (T^2^) = 0.038	
**Pulpal Pathology**	0.000	T^0^-T^1^ = 0.001 T^1^-T^2^ no significative T^2^-T^3^ no significative		Pulpal Pathology & Fracture (T^0^) = 0.000 Pulpal Pathology & Repositioning (T^0^) = 0.000 Pulpal Pathology & Splint (T^0^) = 0.010
